# Thymic Polypeptide Fraction Biomodulina T Decreases Exhausted and Terminally Differentiated EMRA T Cells in Advanced Lung Cancer Patients Treated With Platinum-Based Chemotherapy

**DOI:** 10.3389/fonc.2022.823287

**Published:** 2022-01-27

**Authors:** Gisela María Suárez, Mauricio Catalá, Yadira Peña, Susana Portela, Ana Laura Añé-Kourí, Amnely González, Patricia Lorenzo-Luaces, Manuel Díaz, María de los A. Molina, Karla Pereira, Jenysbel de la C. Hernández, Raúl Ramos, Mary Carmen Reyes, Nuris Ledón, Zaima Mazorra, Tania Crombet, Agustin Lage, Danay Saavedra

**Affiliations:** ^1^ Clinical Immunology Department, Center of Molecular Immunology, Havana, Cuba; ^2^ Oncology Unit, Medical & Surgical Research Center (CIMEQ), Havana, Cuba; ^3^ Pulmonology Hospital “Benéfico Jurídico”, Havana, Cuba; ^4^ Immunology Department, Instituto de Ciencias Básicas y Preclínicas “Victoria de Girón”, Havana, Cuba; ^5^ Biomedical Research Institute, Hasselt University, Hasselt, Belgium; ^6^ Clinical Direction, Centro Nacional de Biopreparados, Bejucal, Cuba

**Keywords:** terminally differentiated T cells, naive T cells, PD-1, non-small-cell lung cancer, biomodulina T, CIMAvax-EGF

## Abstract

Lung cancer is the second cause of cancer related deaths worldwide. Chemotherapy and immunotherapy represent the current standard of care for advanced NSCLC. Platinum-based chemotherapy expands late-differentiated T cell populations. Therefore, immune restoration after chemotherapy to adjuvate the immunotherapeutic potential could be crucial. The aim of this study was to evaluate the effect of Biomodulina T (BT), a thymic polypeptide fraction, on peripheral lymphocytes subpopulations in the context of cancer disease. Additionally, whether these effects might induce a better response to CIMAvax-EGF, an epidermal growth factor (EGF) depleting immunotherapy. Eighteen advanced NSCLC patients were evaluated after being treated with platinum-based chemotherapy. We found that the frequency of terminally differentiated effector T cells re-expressing CD45RA (EMRA) CD4+ (p=0.0031) and CD8+ (p=0.0372) T cells decreased with the administration of BT, whereas CD4+ naive T cells increase in more than 70% of the patients. Remarkably, CD4+ and CD8+ T lymphocytes expressing programmed cell death receptor-1 (PD1) significantly decreased after BT administration (p=0.0005 and p<0.0001, respectively). We also found an enhancement of the anti-EGF antibody response with a large percentage of patients treated with CIMAvax-EGF reaching the good antibody response condition after four vaccine doses. Moreover, the median overall survival of patients treated with CIMAvax-EGF was 16.09 months. In conclusion, our results suggest that the immunorestoration generated by the administration of BT after first-line chemotherapy may induce a better immune response to CIMAvax-EGF that could translate into the clinical benefit of patients diagnosed with advanced NSCLC.

## Introduction

Cancer’s rising prominence is a major health problem around the world. Lung cancer is the leading cause of cancer death in 2020, with an estimated 2.2 million new cases and 1.8 million deaths (1). The most common form of the disease is NSCLC and more than 60% of patients have advanced illness at diagnosis (2).

During the last decade, cancer immunotherapy has been shown to increase survival of patients with advanced cancer (3). Recently, several immunomodulatory drugs, including anti-PD-1 antibodies such as nivolumab and pembrolizumab have shown an improvement in the survival of patients with advanced NSCLC (4–6). However, the molecular heterogeneity of NSCLC makes it more difficult not only for diagnosis but also for the management and therapeutic response in cancer patients (7).

It is widely accepted that epidermal growth factor receptor (EGFR) over-expression in lung cancer cells is associated with tumor progression (8), still in the lack of specific EGFR mutations. In this scenario, its EGF ligand as a poor prognostic factor for individuals with advanced NSCLC (9) became an attractive therapeutic target for lung cancer treatment (10, 11). CIMAvax-EGF, an EGF-depleting immunotherapy, induces neutralizing anti-EGF antibodies which recognize circulating EGF, preventing its binding to EGFR and consequently disrupting the signal transduction cascade associated with proliferation and survival signals in neoplastic cells (12, 13).

Advanced cancer promotes profound immune suppression and hinders an effective antitumor response (14). Additionally, chemotherapeutic treatments influence the immune system by promoting the transition of lymphocytes from less differentiation to late maturation stages, which are sources of pro-inflammatory cytokines supporting systemic inflammation (15). Therefore, immunological restoration in this scenario would enhance the response against cancer (16) and improve the immunotherapeutic potential in cancer patients.

A previous study carried out by our group, presented the effects of BT, a polypeptide thymic fraction, on T cell compartments in older adults with a history of recurrent respiratory infections (17). The administration of BT expanded naive CD4+ T cells, recent thymic emigrants and stem cell-like memory CD8+ T lymphocytes whereas, decreased CD4+ and CD8+ T cells expressing PD1, pointing to its possible anti-exhaustion value in immune response. All this occurred in a context of no expansion of Tregs (17), suggesting BT as a promising immune reconstitution strategy in cancer patients.

In the present study, we recruited patients diagnosed with advanced NSCLC with the aim to evaluate whether the administration of BT immediately after front-line chemotherapy, impacts on the distribution of populations of the immune system. Besides, we explored whether the sequential combination of BT and CIMAvax-EGF influence on the clinical outcome of the patients treated with this novel combination.

## Materials and Methods

### Patients and Treatment

Eighteen patients with histological confirmed stage IIIB or IV NSCLC aged 53 to 82 years were recruited after finished first-line platinum-based chemotherapy (4-6 cycles). Five to seven days after the end of chemotherapy they started treatment with BT: three times per week, during four weeks. Each dose consisted in 3 mg of the polypeptide fraction. The route of administration was intramuscular in the gluteal region. One week after completing BT treatment, the patients started the administration of CIMAvax-EGF vaccine.

**Figure 1:**
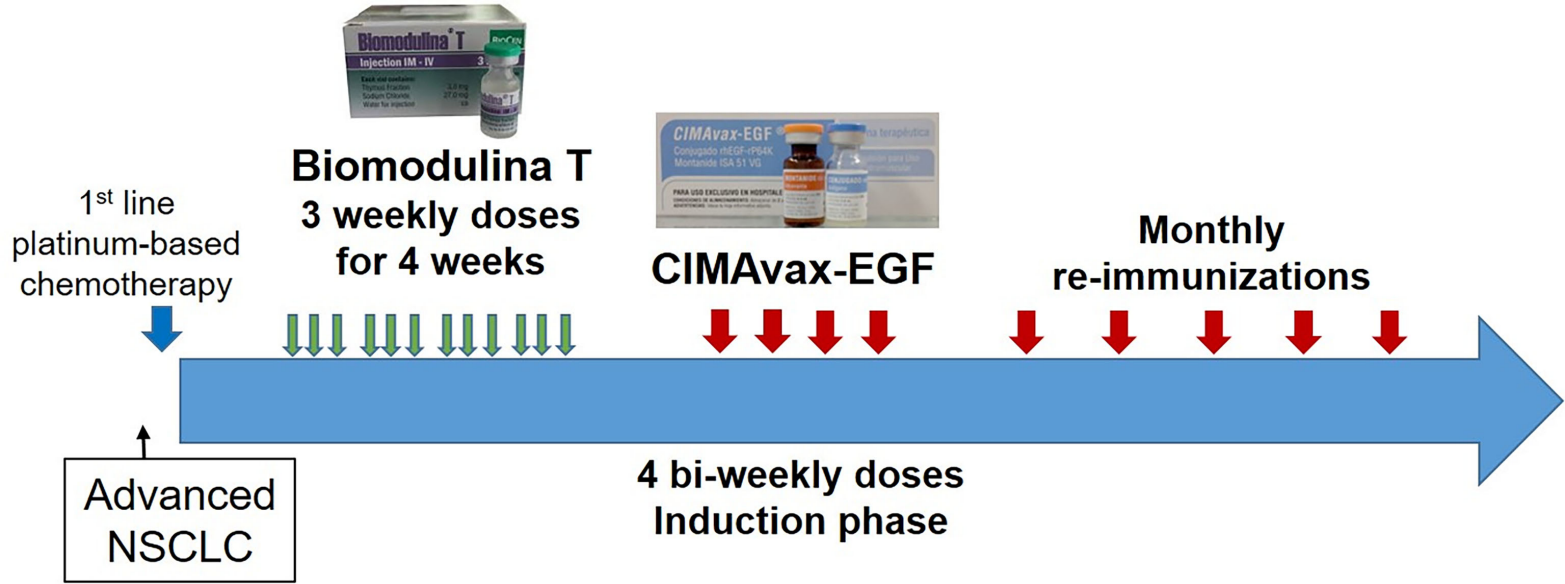
Sequential combination of BT and CIMAvax-EGF in patients diagnosed with advanced Non-small cell lung cancer.

CIMAvax-EGF consists of human recombinant EGF manufactured in yeast (hu-recEGF) chemically conjugated to the P64K *Neisseria meningitides* recombinant protein (reP64K), manufactured in *Escherichia coli*. Both hu-recEGF and P64K were supplied by the Center for Genetic Engineering and Biotechnology, Havana, Cuba. The rhEGF-rrP64K conjugate is stored at 4°C. As adjuvant was employed Montanide ISA 51 VG (NC0962946, Seppic) and emulsified in it, in a proportion 1:1 (v/v) immediately before injection. The vaccine formulation (rhEGF- rP64k/Montanide) was administered at 2.4 mg of total EGF dose per vaccination (divided into four equal 0.6mg intramuscular injections at four sites; two in the deltoids and two in the gluteus), every two weeks for the first four doses (induction period) and then, monthly (**Figure 1**).

The patients were evaluated in the Oncology unit at Medical & Surgical Research Center (CIMEQ) in Havana. The ethics board approved the trial protocol. Informed consent was obtained from each patient before entering in the study. The study was done in compliance with the principles of Good Clinical Practices (according the International Conference of Harmonization) and the Declaration of Helsinki (2013). This study is registered in https://rpcec.sld.cu/en/trials/RPCEC00000358-En the Cuban Public Registry of Clinical Trial (Spanish acronym: RPCEC), a WHO-validated Public Registry, Trial Number RPCEC00000358. Demographic and clinical characteristics are summarized in **Table 1**.

**Table 1:** Demographic and clinical characteristics of patients.

	No	%
n	18	100
Male	12	66.67
Female	6	33.33
Age mean (range)	62.64 (53-80)	–
**ECOG status (0-2)**		
ECOG status 0	5	27.8
ECOG status 1	11	61.1
ECOG status 2	2	11.1
**Histologic subtype**		
Adenocarcinoma	11	61.1
Squamous cell carcinoma	4	22.2
NSCLC	3	16.7
**Response to first-line treatment**		
Complete response	2	11.11
Partial response	4	22.2
Stable disease	11	61.1
Progressive disease	1	5.6
**Evaluation moments/Flow Cytometry**		
After-chemotherapy	18	100
After Biomodulina T	18	100
**Survival Analysis**		
CIMAvax-EGF month 3	18	100

Proportion of patients in each category indicated were calculated of the total absolute number of patients included. ECOG, Eastern Cooperative Oncology Group; NSCLC, Non-small cell lung cancer.

### Processing of PBMC

Blood from patients was collected by venipuncture in heparinized tubes after chemotherapy (Before BT) and at the conclusion of BT (End BT). Peripheral Blood Mononuclear Cells (PBMC) were purified by Ficoll-Paque PLUS centrifugation (Amersham Biosciences). Cells were immediately cryopreserved in RPMI 1640 supplemented with 40% FCS and 10% DMSO until the experiment was performed.

### Measurements of Anti-EGF Antibody Titers and EGF Concentrations

Blood samples were collected at baseline (pre-treatment with CIMAvax-EGF vaccine) and at 3th, 6th, 9th, and 12th months after starting CIMAvax-EGF immunization. Three milliliters of whole blood was spun for 10 min at 3000 rpm to isolate serum. Aliquots of the samples were stored at -80°C until use.

Anti-EGF antibody titers were measured through an enzyme linked immunosorbent assay (ELISA). Recombinant human EGF is immobilized on high binding 96 wells micro-plates. The residual binding sites on the plate are blocked by adding of non-interacting protein [Bovine Serum Albumin (BSA)]. Patients’ sera samples with unknown amount of antibodies are then incubated in all wells and detected by a labeled secondary antibody conjugated with alkaline phosphatase enzyme. Unbound labeled antibody is subsequently removed from all wells by washing, and the solution of diethanolamine with substrate para-nitrophenylphosphate is applied to the plate. The enzyme/substrate reaction provokes a yellow color development and absorbance is measured at 405 nm in an ELISA plate reader after stopping of the reaction (13, 18).

Patients were classified in good antibody responders (GAR) if they developed anti-EGF antibody titers equal or higher than 1:4000. Those vaccinated patients that did not have titers above 1:4000 were classified as poor antibodies responders (PAR) (19, 20).

The serum EGF concentration was measured with a commercial ELISA (UMELISA EGF; SUMA, Centro de Inmunoensayo, Havana, Cuba) (21).

### Flow Cytometry

The anti-human antibodies used were anti-CD3 (RPE-Cy5, MCA463C, Bio-Rad), anti-CD4 (Alexa Fluor 700, clone RPA-T4, Biolegend), anti-CD8 (APC AF 700, clone B4918, Beckman Coulter), CD57 (FITC, clone TB01, Bio-Rad), anti-CD28 (PE, clone CD28.2, BD Pharmingen), anti-CD45RA (PE-CF594, clone H100, BD Horizon), anti-PD1 (PE Cy7, clone J105), anti-CCR7 (APC-Cy7, clone 2043177, Biolegend). The monoclonal antibodies were used for staining in the following panel: CD3 PE-Cy5/CD4 AF-700/CD8 APC-AF-700/CD57 FITC/CD28 PE/CD45RA PE-F594/PD1 PE-Cy7/CCR7 APC-Cy7.

All steps were performed at 4°C. After thawing the cells in RPMI medium and 10% BSA, surface staining was performed using antibodies in FACS buffer (PBS with 5 mM EDTA and 0.2% BSA) in the dark at 4 °C for 30min. Subsequently, cells were washed twice and fixed for 30 min at room temperature (Formaldehyde 2%/PBS 1X/BSA/sodium azide). Data acquisition was performed with a Gallios flow cytometer (Beckman Coulter, 3-laser configuration). The data were processed with FlowJo software [Tree Star Inc., v 10(2)] and data exported as tabulated results for statistical analyses.

### Statistical Analysis

The Shapiro–Wilk normality test was used to determine the normal distribution of peripheral populations. Statistical significance between the groups of two study moments: before BT and at the end of BT administration were evaluated using t-tests and Wilcoxon, for paired values tests, when data passed or failed normality test, respectively. These statistical analyses were performed using GraphPad version 7. The statistical data were considered significant if p < 0.05.

Overall survival (date of first vaccination to date of death or last contact) was estimated by the Kaplan-Meier method. Cox regression was used for survival analysis in relation to the difference between CD4+PD1+ T cell frequencies before and after BT. Groups of patients with high and low CD4+PD1+ lymphocyte T cell difference were determined from the cut-off point previously calculated by Maximally selected log-rank statistics test. Survival and Progression-free Survival comparison between groups were done with a standard log-rank test. In agreement with the methodology proposed by Lambert PC and co-workers (22).

The Spearman correlation coefficient was used to estimate the correlation between EGF concentration and anti-EGF titers.

The statistical system SPSS (version 21) and R version 3.5 were used for statistical analysis.

## Results

### Biomodulina T Decreases the Frequency of EMRA CD4+ and CD8+ T Cells After Chemotherapy

**Figure 2:**
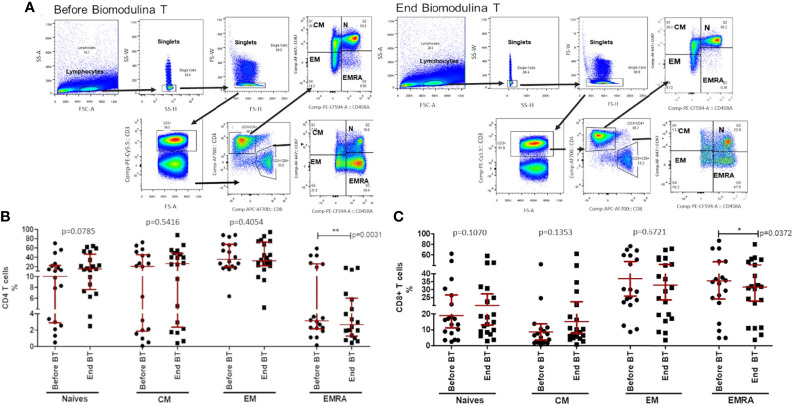
CD4+ and CD8+ T cell subsets before and after the treatment with BT. **(A)** Gating strategy for single CD3+ T cells, then gated on CD4+ and CD8+ T cells and subsets: naive (Naive, CD45RA+CCR7+), central memory (CM, CD45RA−CCR7+), effector memory (EM, CD45RA−CCR7−) and terminally differentiated effector T cells re-expressing CD45RA (EMRA, CD45RA+CCR7−). **(B)** Stages of cell differentiation in CD4+ T lymphocytes after chemotherapy and at the end of BT treatment. **(C)** Stages of cell differentiation in CD8+ T lymphocytes after chemotherapy and at the end of BT treatment. The asterisks indicate statistically significant differences among the groups (*p<0.05; **p<0.01); using Paired t test or Wilcoxon test.

The distribution of naive (CD45RA+CCR7+), central memory (CM, CD45RA-CCR7+), effector memory (EM, CD45RA-CCR7-) and terminally differentiated effector T cells re-expressing CD45RA (EMRA, CD45RA+CCR7-) were explored in CD4+ and CD8+ T cells before starting treatment with BT (one week after finishing first-line platinum-based chemotherapy) and five to seven days after finishing BT administration. The analysis showed a significant decrease in the percentage of EMRA CD4+ T cells at the end of treatment with BT (p=0.0031; Wilcoxon test; **Figures 2A, B**). Regarding CD8+ T cells, a significant reduction of EMRA was also observed after BT administration (p=0.0372, Paired t test; **Figures 2A, C**).

An upward trend of both percentage and absolute count of CD4+ T cells was observed at the end of treatment with the thymic factor, when comparing the two time-points studied (p=0.0785; Wilcoxon test; **Figure 2B**; p=0.0771; Wilcoxon test; data not shown). Even though no significant differences were achieved, an increase of naive CD4+ T lymphocytes was observed 71.43% of patients after the administration of BT. No other subset (i.e, CM or EM) was found to be influenced by BT within the CD4+ and CD8+ T cells compartments when administered after front-line chemotherapy.

### Programmed Death 1 Decrease With Biomodulina T Administration in CD4+ and CD8+T Cells

**Figure 3:**
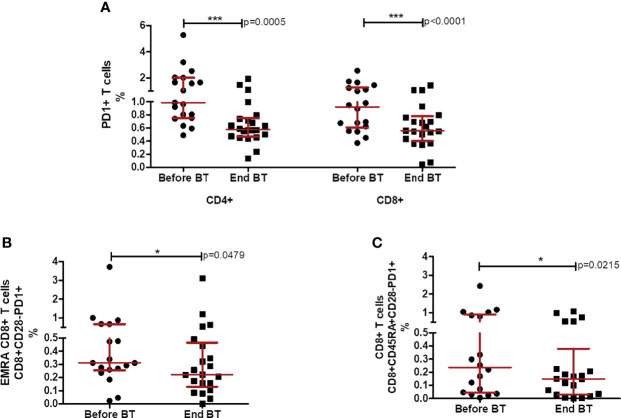
Percentages of CD4+ and CD8+ T cells expressing PD1 before and after the treatment with BT. **(A)** Percentage of PD1+ in CD4 and CD8+ T cells after chemotherapy and at the end of Biomodulina T treatment. **(B)** Relative percentages of CD8+CD28-PD1+ T lymphocytes after chemotherapy and at the end of BT treatment. **(C)** Relative percentages of CD8+CD45RA+CD28-PD1+ T cells after chemotherapy and at the end of BT administration. The asterisks indicate statistically significant differences among the groups (*p<0.05; ***p<0.001) using Wilcoxon test. Before BT, Before treatment with BT; End BT, At the end of BT administration; NSCLC, Non-small cell lung cancer.

PD-1 (Cell Death Protein-Programmed 1) expression has been demonstrated in several tumor types, including NSCLC, suggesting that PD1 receptor is a common molecule used by tumors to escape immune surveillance and sustain tumor growth (23). In the present study, we evaluated the expression of PD1 in CD4+ and CD8+ T cells. The baseline analysis revealed similar levels of PD1 in CD4+ T cells in comparison to CD8+ T lymphocytes of patients diagnosed with advanced NSCLC (**Figure 3A**). Within five to seven days after the last dose of BT, CD4+ (p=0.0005; Wilcoxon test; **Figure 3A**) and CD8+ T cells (p<0.0001; Wilcoxon test; **Figure 3A**) expressing PD1 significantly decreased.

Additionally, we assessed the frequency of PD1 positive cells within terminally differentiated CD4+ and CD8+ T lymphocytes. We found a significant reduction of CD8+CD28-PD1+ T cells (p=0.0479; Wilcoxon test, **Figure 3B**) and CD8+ CD45RA+ CD28-PD1+ T lymphocytes (p=0.0215; Wilcoxon test; **Figure 3C**) at the end of treatment with BT.

### Anti-EGF Antibody Titers After the Sequential Combination of Biomodulina T and CIMAvax-EGF

**Figure 4:**
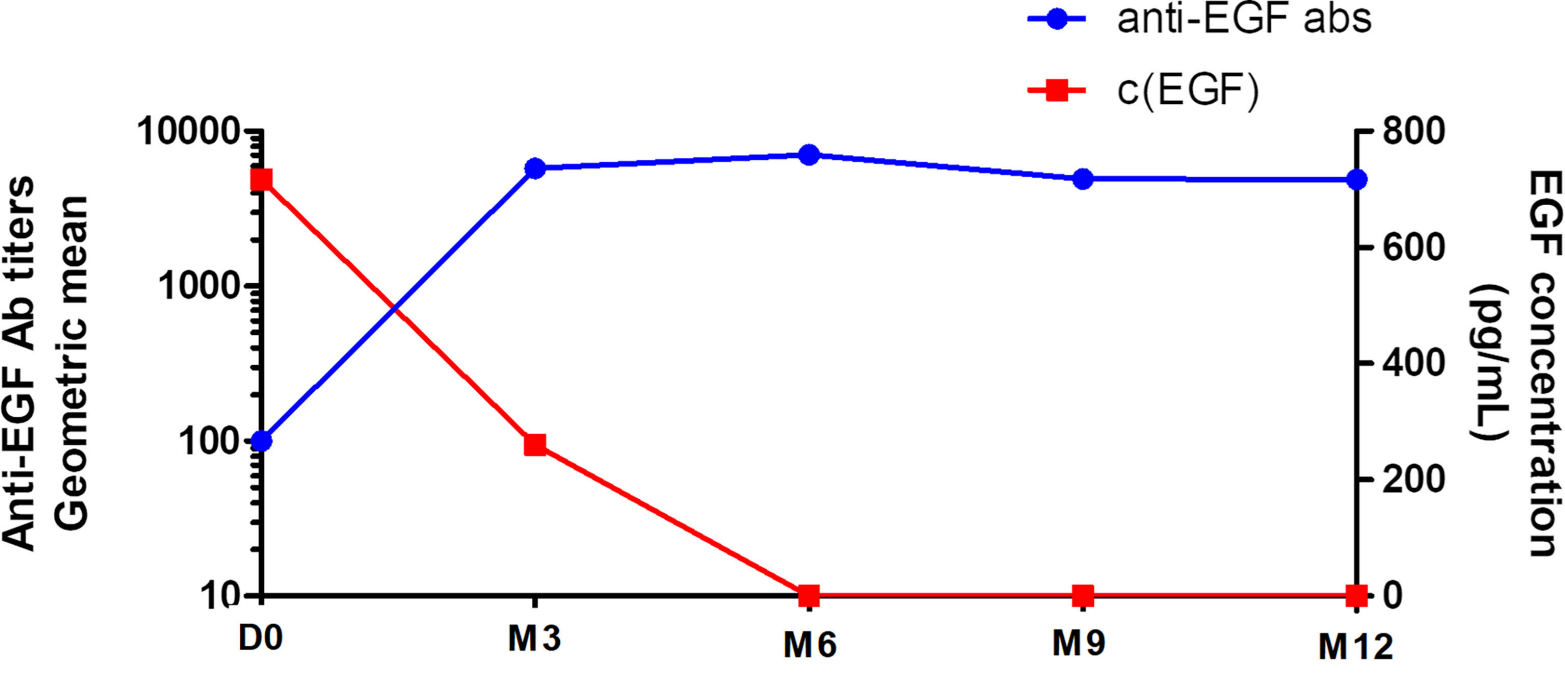
Kinetics of the anti-EGF antibody titers and serum EGF concentration in vaccinated patients. D0, baseline CIMAvax-EGF evaluation; M3, month 3 of evaluation; M6, month 6 of evaluation; M9, month 9 of evaluation; M12, month 12 of evaluation; c, concentration.

With the administration of CIMAvax-EGF immediately after the treatment with BT, anti-EGF antibody titers rose exponentially during the induction phase (bi-weekly first 4 doses), reaching a plateau during the maintenance phase (monthly re-immunizations), as has been previously described for CIMAvax-EGF immunization (19, 20). The geometric mean of anti-EGF antibody titers rose close to 1:6000 of serum dilution from month 3 until month 12 of evaluation (**Figure 4**). Remarkably, the percentage of patients reaching the GAR condition, as they developed anti-EGF antibody titers above 1:4 000 sera dilution, after four vaccine doses was 84.2%. At the sixth month of vaccination, it rose to 93.3%.

As expected, there was a significant inverse correlation between anti-EGF antibody titers and serum EGF concentration after CIMAvax-EGF immunization (r=-0.486; p= 0.048; Spearman test; **Figure 4**). The mean of EGF serum concentration at baseline was 966 pg/ml and the median level was 717 pg/ml. The immunization brought down the EGF serum concentration to almost undetectable levels, as previously described for this EGF depleting immunotherapy (19, 20) (**Figure 4**).

### Survival Benefit in Patients Diagnosed With Advanced NSCLC Who Received Biomodulina T and CIMAvax-EGF Vaccine

**Figure 5:**
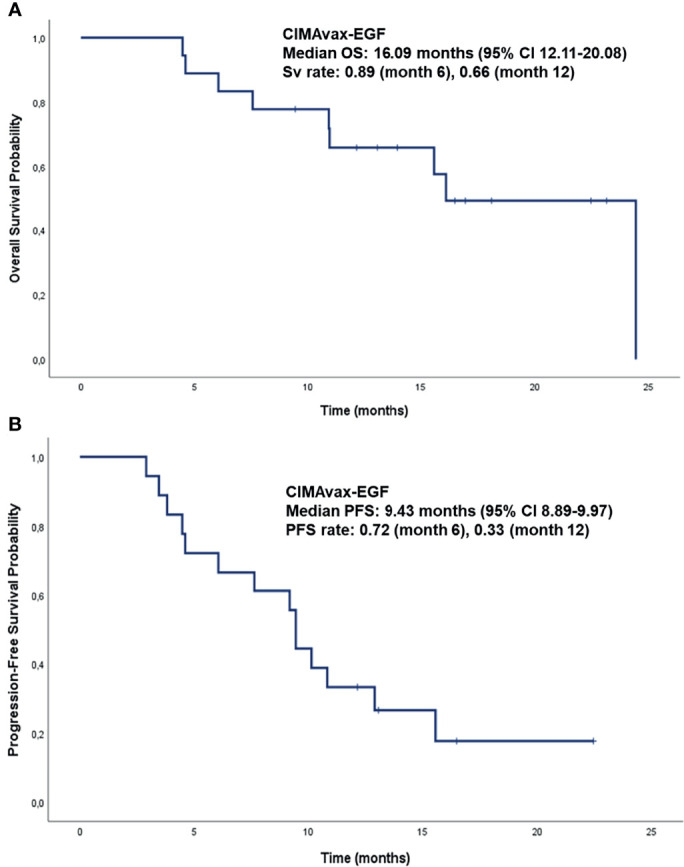
Overall Survival and Progression-Free Survival analysis of patients diagnosed with NSCLC, treated with the sequential combination of BT and CIMAvax-EGF (used as switch-maintenance strategy), after platinum-based chemotherapy. **(A)** Overall Survival of patients diagnosed with NSCLC, treated with BT immediately after platinum-based chemotherapy and then CIMAvax-EGF as switch-maintenance strategy. **(B)** Progression-Free Survival of patients diagnosed with NSCLC, treated with BT immediately after platinum-based chemotherapy and then CIMAvax-EGF as switch-maintenance strategy. OS, Overall survival; PFS, Progression-Free Survival; CI, confident interval.

The sequential combination of BT and CIMAvax-EGF after platinum-based chemotherapy was safe. As previously reported, neither BT nor CIMAvax-EGF were related to grade 3 or 4 adverse events (AE). BT induced no AE, whereas the most frequent related AE reported after CIMAvax-EGF were injection site pain, fever, nausea and headache. No serious AE were observed. The eighteen patients treated with CIMAvax-EGF had a median follow-up time of 16.95 months (95% CI 13.19-20.7 months). The median overall survival (OS) was 16.09 months (95% CI 12.11-20.08 months). OS rates at 6 and 12 months were 89% and 66%, respectively (**Figure 5A**). The median progression-free survival (PFS) was 9.43 months (95% CI 8.89-9.97 months). PFS rates at 6 months was 72% and 33% at 12 months (**Figure 5B**).

**Figure 6:**
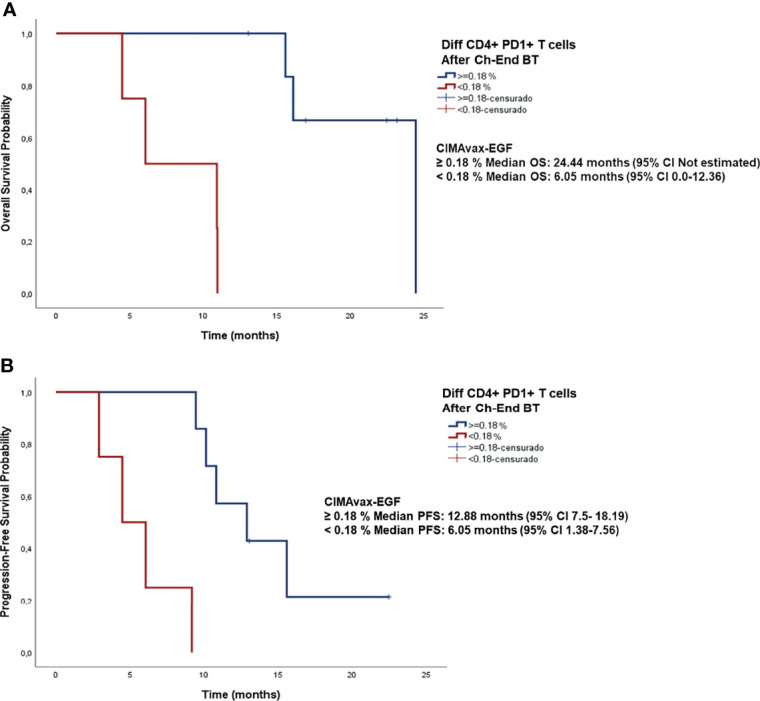
Survival analysis of patients diagnosed with NSCLC stratified according to the difference of the percentage of CD4+PD1+ T cells before and after BT administration. **(A)** Overall survival curves. Median OS for patients with CD4+PD1+ difference >0.18% was 24 months (95% CI not estimated) versus 6 months (95% CI 0.0-12.36 months) for patients with CD4+PD1+ difference <0.18%, p<0.0001 **(B)** Progression-free survival curves. Median progression-free survival time for patients with CD4+PD1+ difference >0.18% was 12.88 months (95% CI 7.5-18.19 months) versus 6.05 months (95% CI 1.38-7.56 months) for patients with CD4+PD1+ difference <0.18%, p<0.0001.

Looking for the clinical relevance of CD4+PD1+ T cell depletion, we focused on the difference between CD4+PD1+ T cell frequencies before and after the administration of BT. We found that as this difference increases, the risk of death decreases (p= 0.046; Cox regression; **Supplementary Figure 1**). Based on Cox regression results, the cut-off point that allowed the identification of patients benefited the most from CIMAvax-EGF was 0.18%. Patients with a difference greater than 0.18% achieved a median survival of 24 months (95% CI not estimated), while those with a difference less than 0.18% had a median survival of 6 months (95% CI 0.0-12.36 months) (**Figure 6A**). In contrast, no significant association was found between the difference of the frequency CD8+PD1+ T cells before and after BT and overall survival (p= 0.203; Cox regression; data not shown).

Likewise, a relationship between progression-free survival and the decrease of CD4+PD1+ T lymphocytes at the end of BT administration was observed (p<0.0001; Cox regression; data not shown). Considering the same cut-off point (0.18%) regarding the difference of this exhausted population at before and after BT, patients with a difference greater than 0.18% had a median progression-free survival of 12.88 months (95% CI 7.5-18.19 months), whereas those with a difference less than 0.18% had a median PFS of 6.05 months (95% CI 1.38-7.56 months) (**Figure 6B**).

## Discussion

Immunotherapy, aiming to boost immune system to control cancer has revolutionized the field of oncology. Cancer immunotherapy has evolved with the advances of several forms of treatment including cancer vaccines, cytokine therapies, adoptive cell transfer and, more recently, immune checkpoint inhibitors (ICI) (24). In a previous work, our group suggested BT, as a promising strategy for immune restoration in elderly patients and for the improvement of immunotherapeutic potential in cancer patients (17). The present research described the effects of BT on several cell populations of the immune system when administered to patients diagnosed with NSCLC, immediately after platinum-based chemotherapy. Additionally, we showed here preliminary results of the sequential combination of the polypeptide fraction BT and CIMAvax-EGF, an EGF-depleting immunotherapy employed as switch-maintenance in NSCLC patients.

We described that terminally differentiated T cells (CD4+ EMRA and CD8+EMRA) decreased after BT treatment. Additionally, CD4+ and CD8+ T cells expressing PD1 were reduced after BT administration, whereas CD4+ naive T cells increased. This translates into an enhancement of the anti-EGF antibody response with a large percentage of patients treated with CIMAvax-EGF reaching the good antibody response condition after four vaccine doses.

Recently, our group reported that chemotherapy significantly reduced the frequency of naive and early-differentiated T cells, as well as increased late-stage T cell populations in patients diagnosed with lung cancer (15). The present study showed that those changes could be modified by BT administration. Late-differentiated T cells have a secreting phenotype known as senescence-associated secretory phenotype (SASP) which support a chronic subclinical inflammation, one of the fundamental properties favoring tumor development (25). In this context, we suggest that reducing terminally differentiated T cells and through them the inflammatory tenor in patients with advanced cancer could improve the immune response against the tumor itself and potentiate the efficacy of other immunotherapies. Additionally, in this work, the decrease of terminally differentiated lymphocytes was accompanied by an expansion of CD4+ naive T lymphocytes in around 70% of patients. Bailur and colleagues reported that naive CD8+ T cells increased and terminally differentiated effector T cells re-expressing CD45RA (TEMRA) CD4+ T cells normalized after 12 months in patients treated with docetaxel and cyclophosphamide (26). In a recent research, conducted in elderly subjects with a history of recurrent respiratory infections and no other chronic diseases, our group showed the ability of BT to significantly increase CD4+ naive T cells (17).

It is widely accepted that the expression of PD-1 in T lymphocytes is associated with exhaustion (25, 27). Ottonello and colleagues reported that higher expression levels of PD-1 on peripheral CD8+ T cells was related with poor overall survival in patients diagnosed with NSCLC (28). In the present study, the treatment with BT significantly reduced CD4+ and CD8+ T cell populations expressing PD1. These findings are in line with a previous report of our group regarding the effects of BT on T cell compartments in elderly, in which this polypeptide factor also induced the decrease of CD4+ and CD8+ cells expressing PD-1 (17), thus highlighting its likely effects countering “exhaustion”.

Due to the heterogeneity of NSCLC, treatment using conventional medications has been challenging. Nevertheless, immunotherapy with anti-PD-1/PD-L1 antibodies has achieved remarkable results in the management of the advanced disease (28). Chemo-immunotherapy combinations are already revolutionizing the paradigm of NSCLC treatment (29). Switch maintenance therapy, using alternative agents that were not administered during induction chemotherapy, is a treatment option for advanced NSCLC. In patients receiving immune checkpoint inhibitors (ICIs) alone or in combination with chemotherapy in first-line, continuation maintenance with pembrolizumab alone or combined with pemetrexed (30, 31), atezolizumab alone, or combined with bevacizumab (32, 33) are the recommended schemes in the adenocarcinoma histology. For the squamous setting, pembrolizumab or atezolizumab monotherapies can be used as maintenance therapies (34). The current standard for advanced NSCLC in Cuba for patients without actionable mutations and EGF serum concentration above 870 pg/ml consists of platinum doublets followed by switch maintenance with CIMAvax-EGF (11). Our proposal for combination in this research was the sequential administration of BT and CIMAvax-EGF, both of them after completed front-line chemotherapy. Data from the CIMAvax-EGF phase III trial as switch maintenance therapy showed that the percentage of patients reaching the GAR condition after four vaccine doses was 56% (20). The results of the present research suggest an enhancement of the antibody response, with a larger percentage of patients with anti-EGF antibody titers higher than 1:4000 in the same evaluation moment.

Median overall survival in the present study was 16.09 months. It compares very favorably with that reported in the CIMAvax-EGF phase III trial (20). Our survival data also compares very favorably with that described by Charalambous and colleagues using pembrolizumab as switch-maintenance strategy (11.3 months) (35). As CIMAvax-EGF is an active immunotherapy, it may require some time after administration to induce the specific anti-EGF immune response. Therefore, it has not been frequent the evaluation of PFS in the clinical scenario. In this occasion, based on the benefits conferred by BT to the immune system, we performed the PFS analysis, resulting in 9.43 months. The administration of pembrolizumab as maintenance therapy showed an immune related PFS of 6.8 months (35), very similar to our data.

High soluble PD-L1 (36) and peripheral CD8+PD-1+ T cells have been associated with negative impact on PFS, OS and ICI-response (28). Remarkably, in this work, patients with a greater decrease in peripheral CD4+PD-1+ T lymphocytes at the end of BT administration had a better survival than those patients with lower decrease of this exhausted population. Therefore, we highlight the clinical relevance of CD4+PD-1+ T cell depletion in this context. Further research will be necessary to confirm its value as a biomarker associated with the clinical outcome.

Our study was conducted in a reduced number of patients. In spite of these limitation, it provides, for the first time, evidences regarding BT as a promising strategy for immune restoration after chemotherapy and to adjuvate immunotherapeutic potential in cancer patients. In summary, BT administered after platinum-based chemotherapy reduce terminally and exhausted CD4+ and CD8+ T cell populations and expands CD4+ naive T lymphocytes. Our results suggest that the sequential combination of BT and CIMAvax-EGF in patients diagnosed with advanced NSCLC after front-line chemotherapy induce a positive clinical evolution of the patients. Further studies need to be done with a larger sample size to confirm the clinical benefits of this novel combination.

## Data Availability Statement

The raw data supporting the conclusions of this article will be made available by the authors, without undue reservation.

## Ethics Statement

The studies involving human participants were reviewed and approved by Medical & Surgical Research Center (CIMEQ) Ethics board. The patients/participants provided their written informed consent to participate in this study.

## Author Contributions

DS, GS, MR, and LA conceptualized and designed the study. MC, YP, SP, DS, MD, and MM recruited and treated the patients. DS, AG, GS, AA-K, and KP contributed to sample collection. GS, AA-K, DS, RR, JH, and KP (immunological assessments). PL-L, GS, and DS performed the statistical analysis. GS, DS, ZM, TC, NL, and AL analyzed and interpreted the data. GS and DS drafted the manuscript. AL, AA-K, ZM, NL, TC, and DS critically revised the manuscript. All authors contributed to manuscript revision, read, and approved the submitted version.

## Funding

This study was supported by the Center of Molecular Immunology (216 St, corner 15, PO box 16040, Atabey, Havana, Cuba).

## Conflict of Interest

The authors declare that the research was conducted in the absence of any commercial or financial relationships that could be construed as a potential conflict of interest.

## Publisher’s Note

All claims expressed in this article are solely those of the authors and do not necessarily represent those of their affiliated organizations, or those of the publisher, the editors and the reviewers. Any product that may be evaluated in this article, or claim that may be made by its manufacturer, is not guaranteed or endorsed by the publisher.

## Supplementary Material

Supplementary Figure 1 Survival curve and 95% confidence interval of the difference between CD4+PD1+ T cell frequencies before and after the administration of BT estimated using Cox regression model. HR, hazard ratio; d.f., degrees of freedom; CI, confident interval.
