# Supplementary figures and images for: Thymic Polypeptide Fraction Biomodulina T Decreases Exhausted and Terminally Differentiated EMRA T Cells in Advanced Lung Cancer Patients Treated With Platinum-Based Chemotherapy
Source: Front Oncol. 2022 Jan 27;12:823287. doi: 10.3389/fonc.2022.823287 (PMC8828575; doi:10.3389/fonc.2022.823287)

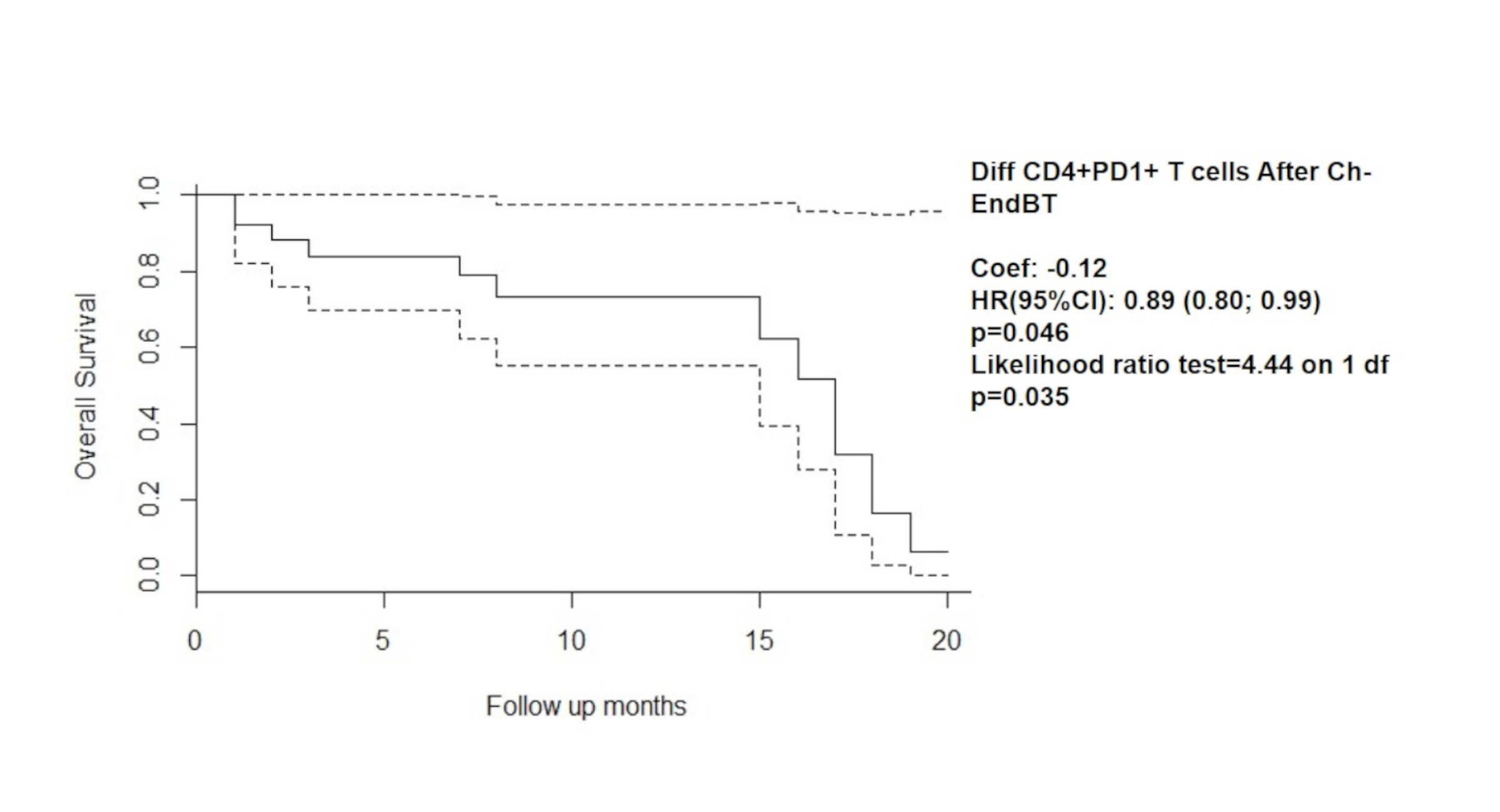

Supplement: Supplementary Figure 1 — Survival curve and 95% confidence interval of the difference between CD4+PD1+ T cell frequencies before and after the administration of BT estimated using Cox regression model. HR, hazard ratio; d.f., degrees of freedom; CI, confident interval. [file Image_1.tif]
